# Enhancing Spectral Reflection through Controlled Phase Distribution Using Doped Polar-Dielectric Metasurfaces

**DOI:** 10.3390/ma13092007

**Published:** 2020-04-25

**Authors:** Mohsen Janipour, Kürşat Şendur

**Affiliations:** Faculty of Engineering and Natural Science, Sabanci University, Istanbul 34956, Turkey

**Keywords:** reststrahlen band, plasmon polariton, phonon polariton, carrier concentration, localized resonance, reflection bandwidth

## Abstract

Controlling the phase distribution of wavefronts using optical metasurfaces has led to interesting optical properties and applications. Here, we explore the control of phase distribution through polar-dielectric metasurfaces composed of doped SiC nanosphere arrays. We investigate the impact of doping concentration on the optical properties of SiC nano-spheres. Our results indicate that increasing the doping of SiC nanoparticles influenced electric dipolar resonances, whereas it did not change the dipolar resonances. Using this concept, we numerically studied the extension of this idea to form metasurface arrays of single, dimer and linear trimers of the doped SiC nano-spheres. Using different doping schemes, we studied the improvement of the reflectivity at frequencies greater than the longitudinal optical phonon frequency.

## 1. Introduction

Optical metasurfaces, composed of metallic resonators, demonstrate exotic optical properties such as bandpass [1,2] bandstop [3,4,5,6,7] frequency selection, right/left handed circular polarizability [8,9,10,11,12], linear to circular conversion [13,14,15,16], conical beam patterning [17,18,19], focused transmission and reflection [20,21,22,23] and flat lensing behaviors [24,25,26,27]. In addition to metasurfaces composed of metallic nanostructures, similar properties have been shown for metasurfaces composed of silicon nanoparticles [27]. The primary goal of these periodic structures is to manipulate the wavefront of the metasurfaces by patterning and shaping the nanoparticles around their resonance frequencies. In other words, controlling the phase of light using the metasurface’s resonances, one can engineer the wavefront and therefore, interesting optical properties can be achieved. By modifying and improving the conventional Snell’s law, Yu et al. [28] and Ni et al. [29] have shown that the gradient of phase discontinuity at the interface of two mediums with different dielectric function can engineer the direction of the reflected and transmitted light. The idea of the phase discontinuity gradient has been established based on the phase shift of the nanoparticle resonators which can be realized through shaping the nanoparticles. One well-known conventional method of shaping nanoparticles is elongation in which the nanoparticles with the same material properties can resonate at different localized surface plasmon resonance (LSPR) frequencies [30].

In the literature, Zetterling et al. [31] have shown that due to their mechanical tolerability and thermal stability the polar dielectrics (especially SiC), are excellent for high temperature and pressure operations such extreme environments applications. Polar dielectric materials, like SiC [32] and GaN [33] can present negative permittivity properties in the infrared (IR) regime between the longitudinal (LO) and transverse (TO) optical phonon frequencies known as the Reststrahlen band [34,35,36,37]. Moreover, it has been shown that an optically small spherical nanoparticle can support the localized surface vibrational phononic mode which is known as the localized surface phonon resonant (LSPhR) mode [37]. In polar dielectrics, or ionic crystals, the LSPhR mode happens in the Reststrahlen band [30]. The scattering cross-section resonance of nanoparticles composed of polar dielectrics is sharp, which is attractive for controlling the phase of light using nanoparticle surfaces.

The idea of controlling the phase fronts is well known by changing the LSPR of metallic nanoparticles through shaping them. While this idea has been explored in plasmonic [21] and dielectric [27] metasurfaces, it has not been studied in the THz regime with thermomechanically stable materials, such as SiC. The use of thermomechanically stable materials is crucial in extreme environment applications because of their attractive properties such as a high melting point and high durability against stress. In this manuscript, we show control of the phase fronts using SiC, a thermomechanically stable material. In the literature, phase control has been achieved through shaping the materials into desired forms. In this manuscript, we propose an alternative method to phase control through doping of the nanoparticles, which can be more suitable for materials such as SiC. We propose, instead of shaping the SiC nanoparticles to control phase fronts, a novel method of increasing the carrier concentrations of spherical nanoparticles. Using this phase control, we demonstrate that high reflection can be achieved using SiC surfaces. This finding can be particularly attractive for the use of SiC in extreme environments with high reflection. In this paper, the improvement of reflection for SiC for frequencies greater than $\omega_{LO}$ is also shown. It is well known that reflection at the air-SiC surface is substantially reduced for frequencies greater than $\omega_{LO}$ [37]. In this work, we show that by tailoring the phase distribution of a metasurface, the reflection spectrum can be improved for frequencies greater than $\omega_{LO}$.

In this study, using Mie theory and according to the interaction of the plasmons and the optical phonon vibration of the crystalline nanoparticles composed of polar dielectrics, first we show that the localized surface resonances can be controlled by doping a spherical nanoparticle. We selected the size of the nanoparticles as small enough to excite the dipolar modes. As a result, the effects of carrier concentration on the higher order modes are not studied in this manuscript. In this study, using the finite-difference time-domain (FDTD) method, we investigated the interaction of the plasmons and the optical phonon vibration of the crystalline nanoparticles composed of polar dielectrics. We show numerically that localized surface resonances can be controlled by doping a spherical nanoparticle. We extend this idea to achieve enhanced spectral reflection of IR radiation using metasurface arrays of the single, dimer and linear trimers of the doped SiC nano-spheres. Using different doping schemes, we study the improved reflectivity at the frequencies greater than the longitudinal optical phonon frequency.

## 2. Doping Effects on the SiC Nanoparticles

**Figure 1:**
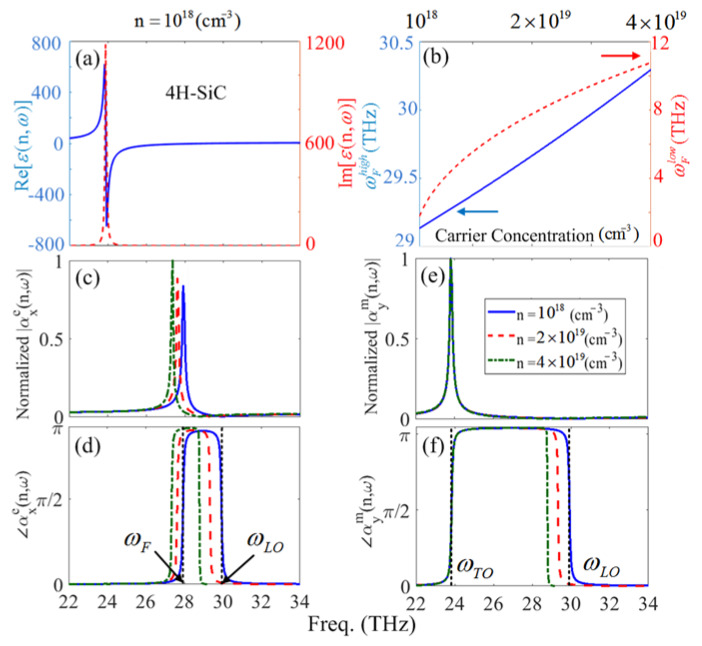
(**a**) The real and imaginary parts of the dielectric function of SiC, (**b**) The Frölich frequencies $\omega_{F}^{low}$ (dashed curve) and $\omega_{F}^{high}$ (solid-curve) for a 4H-SiC nanoparticle using Equation (1) and Equation (2) versus doping concentration, respectively. (**c**–**f**) The normalized electric and magnetic polarizability amplitudes and phase for n = $10^{18}\left(cm^{-3}\right)$ (solid curve), n = $2\times 10^{19}\left(cm^{-3}\right)$ (dashed curve), n = $4\times 10^{19}\left(cm^{-3}\right)$ (dashed-dotted curve), respectively.

In this section, we investigate the doping effects on the optical response of the sub-wavelength nanoparticles composed of 4H–SiC and their individual LSPhR frequency shift. Recently, it has been shown that increasing the number carriers in a bulk 4H-SiC and GaN medium can present a wider Reststrahlen band by enhancing the LO resonant frequency [38,39]. The impact of the optical phonon resonances in the complex dielectric function of the polar-dielectrics can be modeled by the classical Lorentzian resonance function and the effect of doped carriers can be added to the Lorentzian function as a Drude term. With this approach, the dielectric function of SiC can be represented as the summation of the Lorentzian and Drude function [33,40,41]:

(1) $$\varepsilon \left(n,\omega \right)=\varepsilon_{\infty }\left[1+\frac{\omega_{LO}^{2}-\omega_{TO}^{2}}{\omega_{TO}^{2}-\omega (\omega +i\Gamma )}\right]-\frac{\omega_{p}^{2}\left(n\right)}{\omega (\omega +i\gamma )}$$

where, $\varepsilon_{\infty }$ = 6.5 is the high-frequency permittivity constant, $\omega_{LO}\approx$29 THz and $\omega_{TO}\approx$ 24 THz are the longitudinal and transverse optical phonon frequencies, respectively, with the damping frequency of Γ≈0.08 THz [34,42]. The second term of Equation (1), the plasmon frequency, is related to the carrier concentration through $\omega_{p}\left(n\right)=\sqrt{4\pi ne^{2}/m^{*}}$ with carrier effective mass of $m^{*}=0.36m_{0}$ where $m_{0}$ is the electron mass. In Equation (1) the carrier collision frequency is related to the carrier mobility and the effective mass through $\gamma \left(n\right)=e/m^{*}\mu \left(n\right)$ with $\mu \left(n\right)\approx 4.8\times 10^{9}n^{-0.4}$ $cm^{2}V^{-1}s^{-1}$ [38]. Figure 1a depicts the real (solid-curve), and imaginary (dashed curve) parts of the dielectric function for 4H–SiC with the carrier concentration of *n* = $10^{18}$ $\left(cm^{-3}\right)$. As mentioned earlier, the shaping of the metallic nanoparticles to oblate and prolate nano-spheroids, nanorods and V-shapes is a well-investigated method for changing the LSPR frequency [28]. This approach, however, is more suitable for nanoparticles composed of soft metals, such as Au and Ag. For use in extreme environment applications, materials with higher thermomechanical tolerance are desired which normally possess high melting point. Among these materials, polar dielectrics such as SiC are particularly attractive due to their optical response properties. These optical properties are especially interesting in the IR regime, where a sub-wavelength SiC nanoparticle can demonstrate an optically resonant response.

For a spherical nanoparticle with a sub-wavelength size and immersed in air the resonant condition known as the Frölich frequency is only satisfied if Re[ε(n,ω)] = −2 [43]. The Frölich resonant condition arises from the static polarizability formula of $\alpha_{0}^{e}\propto$ [ε(n,ω)−1]/[ε(n,ω)+2] that governs for small spheres in the quasi-static approximation regime. Thus, for a doped spherical SiC nanoparticle with a constant radius (for instance r= 250 nm) it can be shown that two Frölich frequencies [i.e., $\omega_{F}^{low}\left(n\right)$ and $\omega_{F}^{high}\left(n\right)$] at separate frequency bands can be obtained:

(2) $$\omega_{F}^{low/high}\left(n\right)={\left[\frac{\xi \left(n\right)\mp \sqrt{\xi^{2}\left(n\right)-4\varepsilon_{\infty }\omega_{TO}^{2}\left[\omega_{p}^{2}\left(n\right)-2\right]}}{2\varepsilon_{\infty }}\right]}^{\frac{1}{2}}$$

where, $\xi \left(n\right)=\omega_{p}^{2}\left(n\right)+\varepsilon_{\infty }\omega_{LO}^{2}-2$. The latter issue is in contrast with the resonant plasmonic nanoparticles composed of noble metals in which the spectral position of the LSPR frequencies can be varied through the shape factors.

According to Equation (2), for the pure SiC nanoparticle (i.e.,n=0), the only resonant frequency is due to the optical phonon resonances—or in other words $\omega_{F}^{high}$—which is spectrally limited between $\omega_{TO}<\omega_{F}^{high}<\omega_{LO}$. In addition, according to Equation (2), it is expected that the appearance of $\omega_{F}^{low}$ is directly related to the carrier concentration and consequently, the individual plasmon frequency. Figure 1 shows the $\omega_{F}^{low}$ (dashed curve) and $\omega_{F}^{high}$ (solid-curve) versus doping concentration for a SiC nano-sphere, respectively.

In Figure 1b, it can be seen that for the SiC nano-sphere and the considered doping concentration, the impact of the increasing doping results in a wide region of frequency variation for $\omega_{F}^{low}$ however; the carrier concentration enhancement leads to small spectral variations for $\omega_{F}^{high}$. Here, $\omega_{F}^{low}$ is affected by the increase of the plasmon frequency as a result of increased doping; and small red-shift of $\omega_{F}^{high}$ is due to the plasmon-phonon interactions caused by increasing the doped carrier concentration which means that the energy of the phononic part is increased due to higher collisions of extra carriers [38,44,45]. However, as it can be expected, enhancing the number of carriers leads to the blue-shift of the $\omega_{F}^{low}$ which in fact represents the higher plasmon frequency. Thus, this tunable localized surface resonance mode of the doped SiC nano-sphere can be considered as a localized surface plasmon/surface phonon (LSP/SPh) resonance. Using the extinction and scattering cross-section spectrums obtained from the Mie theory, $\omega_{F}^{low}$ cannot represent an individual resonant peak in comparison with $\omega_{F}^{high}$. Thus, in the rest of the manuscript we focus on the tunable aspects of $\omega_{F}^{high}.$ For an incident TM-mode of electromagnetic field with $E_{\mathrm{inc}}$ = $E_{0}e^{-i\omega t}$ $a_{x}$ and $H_{\mathrm{inc}}$ = $H_{0}e^{-i\omega t}$ $a_{y}$, the electric and magnetic polarizabilities of a SiC nanoparticle are given as [30]:

(3) $$\alpha_{x}^{e}\left(n,\omega \right)=\frac{6\pi i}{k_{0}^{3}}a_{1}\left(n,\omega \right),\;\alpha_{y}^{m}\left(n,\omega \right)=\frac{6\pi i}{k_{0}^{3}}b_{1}\left(n,\omega \right)$$

where, $k_{0}=2\pi /\lambda_{0}$ is the wavenumber in free space, $a_{1}\left(n,\omega \right)$ and $b_{1}\left(n,\omega \right)$ are the carrier concentration dependent relation of the dipolar term of the scattering Mie coefficients. Using the presented dielectric function of SiC from Equation (1), the scattering Mie coefficients for all of the stimulated multipole electric polarizabilities can be presented as:

(4) $$a_{l}\left(n,\omega \right)=\frac{m\left(n,\omega \right)\psi_{l}\left[m\left(n,\omega \right)k_{0}r\right]{\psi^{\prime }}_{l}\left(k_{0}r\right)-\psi_{l}\left(k_{0}r\right){\psi^{\prime }}_{l}\left[m\left(n,\omega \right)k_{0}r\right]}{m\left(n,\omega \right)\psi_{l}\left[m\left(n,\omega \right)k_{0}r\right]{\xi^{\prime }}_{l}\left(k_{0}r\right)-\xi_{l}\left(k_{0}r\right){\psi^{\prime }}_{l}\left[m\left(n,\omega \right)k_{0}r\right]}\;b_{l}\left(n,\omega \right)=\frac{\psi_{l}\left[m\left(n,\omega \right)k_{0}r\right]{\psi^{\prime }}_{l}\left(k_{0}r\right)-m\left(n,\omega \right)\psi_{l}\left(k_{0}r\right){\psi^{\prime }}_{l}\left[m\left(n,\omega \right)k_{0}r\right]}{\psi_{l}\left[m\left(n,\omega \right)k_{0}r\right]{\xi^{\prime }}_{l}\left(k_{0}r\right)-m\left(n,\omega \right)\xi_{l}\left(k_{0}r\right){\psi^{\prime }}_{l}\left[m\left(n,\omega \right)k_{0}r\right]}$$

with m(n,ω) = $\sqrt{\varepsilon \left(n,\omega \right)}$, $\psi_{1}\left(k_{0}r\right)$ = $k_{0}r$ $j_{1}\left(k_{0}r\right)$, and $\xi_{1}\left(k_{0}r\right)$ = $k_{0}r$ $h_{1}^{\left(1\right)}\left(k_{0}r\right)$, respectively. In Equation (4), $j_{1}$ and $h_{1}^{\left(1\right)}$ demonstrate the spherical Bessel functions of the first and third kind, respectively, where the prime shows their individual argument-based derivatives of the Bessel functions. Figure 1c–f show the normalized amplitude of the electric and magnetic polarizabilities and their relevant phase variations for *n* = $10^{18}$ (solid-curve), *n* = $2\times 10^{19}$ (dashed curve), *n* = $4\times 10^{19}$ $\left(cm^{-3}\right)$ (dashed-dotted curve), respectively. Based on Figure 1c,e, increasing the doping value leads to a red-shift for the $\alpha_{x}^{e}\left(n,\omega \right)$ (or in other words LSPhR); however, increasing the carrier concentration does not affect the $\alpha_{y}^{m}\left(n,\omega \right)$ resonant frequency. On the other hand, since $\omega_{F}$ is located in the Reststrahlen band which can be controlled by increasing the carrier concentration, enhancement of the dopant amounts in the SiC nanoparticle (which results in stronger plasmon-phonon interactions) can influence the electric dipolar resonant frequency. In contrast, since the magnetic dipole resonance is located at the $\omega_{TO}$ frequency, which is the pole of the dielectric function, it does not experience an effect by increasing the doping amounts. Based on the results in Figure 1d–f, the phase of the resonating dipoles is varied two times at the resonance frequencies, i.e., $\omega_{F}$ and $\omega_{TO}$; and at $\omega_{LO}$ for the electric and magnetic dipoles, respectively, which is in contrast with the noble metals’ nanoparticles. Although the first phase shifts at $\omega_{F}$ and $\omega_{TO}$ are due to the resonance conditions, the second ones that occur at $\omega_{LO}$ are because of negative permittivity to positive permittivity transition at this frequency.

**Figure 2:**
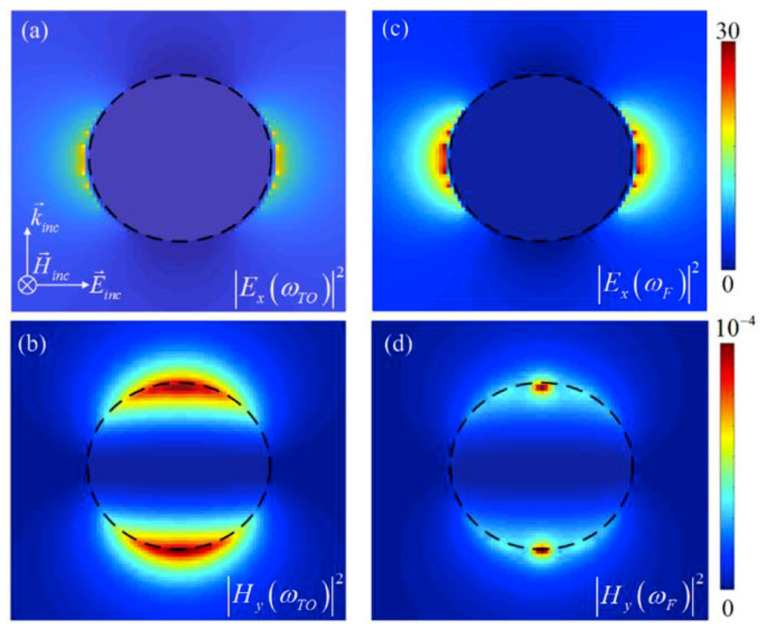
(**a**,**c**) The electric and (**b**,**d**) magnetic field intensities at $\omega_{TO}$ = 24 and $\omega_{F}$ = 28 THz for a 4H-SiC nanoparticle, respectively, for n = $10^{18}$ $\left(cm^{-3}\right)$.

Figure 2a–d illustrates the electric and magnetic field intensities for a carrier concentration of *n* = $10^{18}$ at $\omega_{TO}$ = 24 THz and $\omega_{F}$ = 28 THz, respectively. Based on the results in Figure 2a,c, it can be seen that the electric field intensity at $\omega_{TO}$ is negligible, however it plays an important role at $\omega_{F}$. 

**Figure 3:**
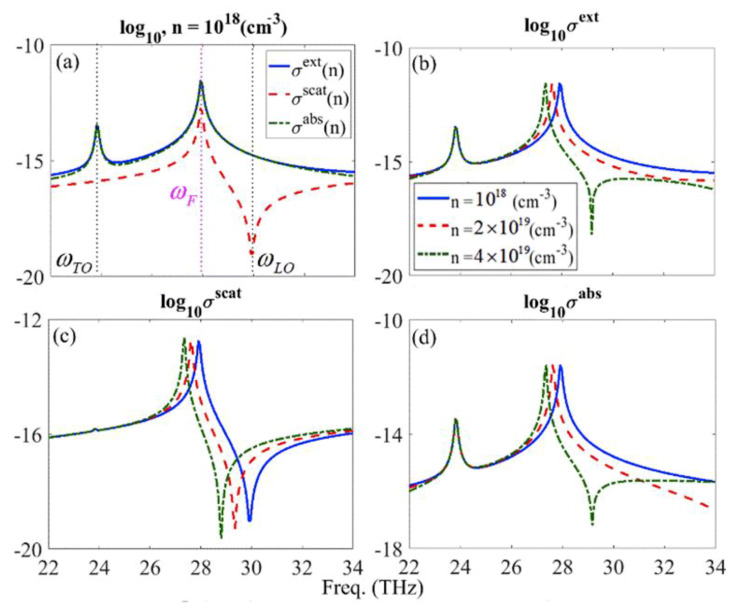
(**a**) The logarithmic carrier concentration dependency of the extinction (solid-curve), scattering (dashed curve) and absorption (dashed-dotted curve) spectrum, respectively, of a 4H-SiC nano-sphere and for *n* = $10^{18}\left(cm^{-3}\right)$. (**b**–**d**) The logarithmic scale of the extinction, scattering and absorption spectrums for *n* = $10^{18}$ (solid curve), *n* = $2\times 10^{19}$ (dashed curve), *n* = $4\times 10^{19}\left(cm^{-3}\right)$ (dashed-dotted curve), respectively.

This indicates that the radiation portion of the corresponding electric dipole in the Poynting vector at $\omega_{TO}$ can be neglected. In contrast, the results in Figure 2b,d reveal that the intensity of the induced magnetic field intensity at $\omega_{F}$ is negligible in comparison to $\omega_{TO}$, which points to the prevailing role of the magnetic dipolar behavior of the nanoparticle at $\omega_{TO}$. These results and observations based on the numerical calculations support the previous discussions based on the theoretical results of the dipolar properties of the considered SiC nanoparticle. To further investigate the doping effects on the optical properties of the SiC nanoparticle in the IR frequencies, we study this impact on the extinction, scattering and absorption cross-sections [i.e., $\sigma^{ext}\left(n\right)$, $\sigma^{scat}\left(n\right)$ and $\sigma^{abs}\left(n\right)$] of the intrinsic and doped SiC particles [30]. Figure 3a demonstrates the logarithmic scale of the extinction (solid-curve), scattering (dashed curve) and absorption (dashed-dotted curve) cross-sections of the SiC nano-sphere with a constant radius of r= 250 nm and carrier amount of *n* = $10^{18}$ $\left(cm^{-3}\right)$, respectively. In Figure 3a, it can be seen that for the SiC nanoparticle, the scattering cross-section is smaller than the extinction and absorption cross-sections and therefore, most of the incident optical energy is absorbed in the nanoparticle. This effect is due to the high imaginary part of the dielectric function of the SiC medium which results in a smaller portion of scattering in the optical extinction cross-section. Although the dominant effect of the electric dipole resonance (i.e.,$\omega_{F}$) is clear in all of the cross sections, the negligible magnetic dipole influence can be tailored in the $\sigma^{ext}$ and $\sigma^{abs}$; and minimum scattering can be achieved at $\omega_{LO}$. In addition, Figure 3b–d shows the carrier dependency of the extinction, scattering and absorption cross-sections spectrums for the doped SiC nanoparticle in the logarithmic scale for *n* = $10^{18}$ (solid curve), *n* = $2\times 10^{19}$ (dashed curve), *n* = $4\times 10^{19}$ $\left(cm^{-3}\right)$ (dashed-dotted curve), respectively.

In Figure 3b–d, it can be seen that the resonant peak of the electric dipole and the relevant dip of the LO phonon resonances are red-shifted by increasing the carrier concentration. This red shift is due to the plasmon-phonon interaction mechanism which can be stronger by increasing the amount of the carrier concentration. In other words, the higher amount of the carrier concentration, can make this interaction strong enough so that it leads to the red shift of the phonon resonance.

## 3. Patterned Surface of the Doped SiC Nanoparticles

The real part of the dielectric function of SiC demonstrates a small-positive value for frequencies greater than $\omega_{LO}$, which in turn results in a small reflection spectrum of the bulk SiC. In addition to the bulk medium, the same mechanism governs the SiC nano-particles. This can be seen from Figure 3a,c where the minimum scattering cross-section occurs at $\omega_{LO}$. In this section, using substrate-induced (SI) metasurfaces composed of the doped SiC nanoparticles, we use a periodic configuration which can compensate for the reflection spectrum for frequencies equal to and/or greater than the LO phonon resonances.

Using the idea of different signs of permittivities for the resonant nanoparticle and substrate, Albooyeh et al. [46] have theoretically and numerically demonstrated that supposing a substrate with a high dielectric permittivity constant (i.e., $\varepsilon_{r}\approx$12) can satisfy the necessary boundary conditions to achieve an artificial perfect electric conductor (APEC). Since in the Reststrahlen band, SiC represents the negative dielectric properties and as mentioned in the previous section, the individual free-standing nanoparticles can be treated as resonant dipoles (Figure 1b,c).

However, satisfying the APEC condition using a high-index substrate can affect the electric polarizability behavior through the induced current density to the substrate; so that the substrate can be replaced by the dipolar image of the nanoparticle. Each single unit-cell consisting of the electric and its induced image can be assumed as an effective dipole ${\tilde{\alpha }}_{x}^{e}\left(n,\omega \right)$. We note that a substrate with $\varepsilon_{r}=$ 14 can provide an APEC in the IR frequencies. Figure 3a,b shows the normalized amplitude and phase of the effective dipole for $n=10^{18}(cm^{-3})$ (solid curve), $n=2\times 10^{19}$ $\left(cm^{-3}\right)$ (dashed curve) and $n=4\times 10^{19}(cm^{-3})$ (dashed-dotted curve), respectively.

**Figure 4:**
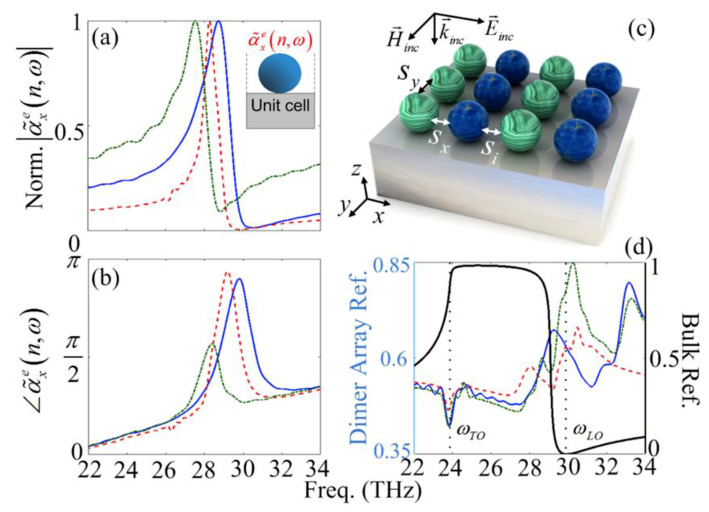
(**a**,**b**) The normalized amplitude and phase of the effective susceptibility of a substrate induced doped SiC nanoparticles for $n=10^{18}$ (solid curve), $n=2\times 10^{19}$ (dashed curve) and n= $4\times 10^{19}$ $\left(cm^{-3}\right)$ (dashed-dotted curve), respectively. (**c**) Schematic representation of the square patterned metasurface composed of SiC nano-spheres dimers with different doping values. (**d**) The reflection spectrum of the dimer arrays for $n_{1}=10^{18}$ and $n_{2}=2\times 10^{19}$ (solid curve), $n_{1}=10^{18}$ and $n_{2}=4\times 10^{19}$ [dashed-curve], $n_{1}=2\times 10^{19}$ $\left(cm^{-3}\right)$ and $n_{2}=4\times 10^{19}$ $\left(cm^{-3}\right)$ [dashed-dotted-curve], respectively, with s= 200 nm.

Similar to the free-standing SiC nanoparticles with the same size (Figure 1b,c); ${\tilde{\alpha }}_{x}^{e}\left(n,\omega \right)$ resonance frequency is red-shifted by increasing the carrier concentration and despite the substrate impacts (especially on phase), the unit-cell shows dipolar behavior. Figure 4c is a schematic of the array of SiC nanoparticle SI dimers with different doping values in a square lattice arrangement with a constant relative permittivity of $\varepsilon_{r}=$14 for the substrate. The separation distance between the dimer unit-cells and their neighbors in the *x*- and *y*-directions; and the dimer internal distance of the nanoparticles are considered equal, i.e., $s_{x}=s_{y}=s_{i}=s$ = 200 nm. Figure 4d shows the relevant reflection spectrum of the SI array with $n_{1}=10^{18}$ and $n_{2}=2\times 10^{19}$ (solid curve), $n_{1}=10^{18}$ and $n_{2}=4\times 10^{19}$ (dashed curve), $n_{1}=2\times 10^{19}$ $\left(cm^{-3}\right)$ and $n_{2}=4\times 10^{19}$ (dashed-dotted curve), respectively. It should be noted that $n_{i}$ with *i* = 1, 2; symbolizes the doping concentration at each nanoparticle of the dimer. In Figure 4d, the black-solid-curve [the right axis] shows the reflection spectrum of the bulk SiC and the vertical dotted-lines elucidate the spectral position of $\omega_{TO}$ and $\omega_{LO}$ frequencies, respectively. According to Figure 4d, it can be seen that a valley of reflection happens for all doping concentrations at $\omega_{TO}$ frequency. In comparison with the reflection spectrum of bulk SiC in which at $\omega_{LO}$ frequency the reflection is around zero, for the arrays of SiC nanoparticles it can be seen that a maximum reflection with amplitude of around 40–60 percent can be achieved. To investigate this structure, we used a full wave analysis of the structure based on three dimensional FDTD method with uniform unit cells of Δx=Δy=Δz = 10 nm by means of the commercial software Lumerical [47]. An incident *x*-polarized plane-wave illuminates the structure along the *z*-axis (top-side). The Bloch periodic boundary condition along the *x*-axis, the symmetric boundary condition along the *y*-axis (in order to provide the square-lattice), and the stretched coordinate perfectly matched layers (PMLs) with 32 layers along the *z*-direction were applied to the computation region. The simulation time was set to 80,000 (fs) to ensure convergence of the results. To achieve better insight into the reflection spectrum properties, we studied the near-field dipolar interaction of the nanoparticles.

**Figure 5:**
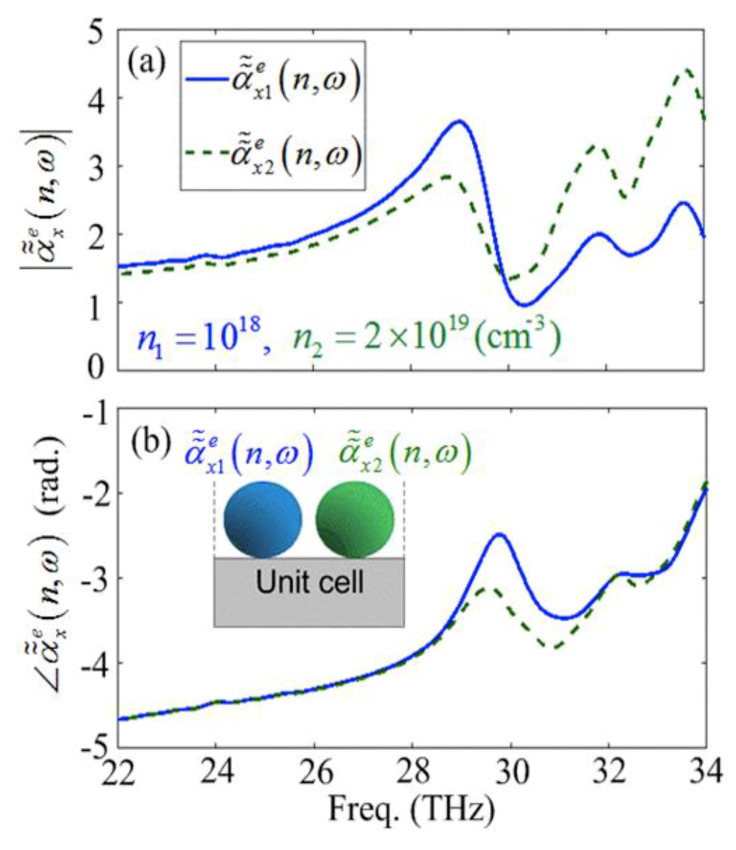
The amplitude and phase of the effective electric susceptibilities of the square patterned array of the SiC dimers [(**a**,**b**)] for $n_{1}=10^{18}$ and $n_{2}=2\times 10^{19}$ $\left(cm^{-3}\right)$ with ${\tilde{\tilde{\alpha }}}_{x1}^{e}\left(n,\omega \right)$ [solid-curve], and ${\tilde{\tilde{\alpha }}}_{x2}^{e}\left(n,\omega \right)$ [dashed-curve], respectively.

Figure 5a,b shows the amplitude and phase of the effective electric susceptibilities of the doped SiC dimer square array for the $n_{1}=10^{18}$ (solid curve) and $n_{2}=2\times 10^{19}$ $\left(cm^{-3}\right)$ (dashed curve), respectively. The inset of the Figure 5b shows the unit-cell arrangement of the considered structure. It can be seen that the first (low-frequency) and the second (high-frequency) modes of the individual reflection spectrum (see Figure 4d) occur at the phase differences of $\Delta \varphi =∠{\tilde{\tilde{\alpha }}}_{x1}^{e}\left(n,\omega \right)-$ $∠{\tilde{\tilde{\alpha }}}_{x2}^{e}\left(n,\omega \right)\approx$ 0.7(rad.) and 0 (rad.), respectively, while the relevant valley occurs at Δφ≈ 0.35 (rad.). Furthermore, it can be interpreted that the first mode happens near the resonant frequency of the single nanoparticles which are slightly red-shifted due to the phononic interactions between the nanoparticles.

**Figure 6:**
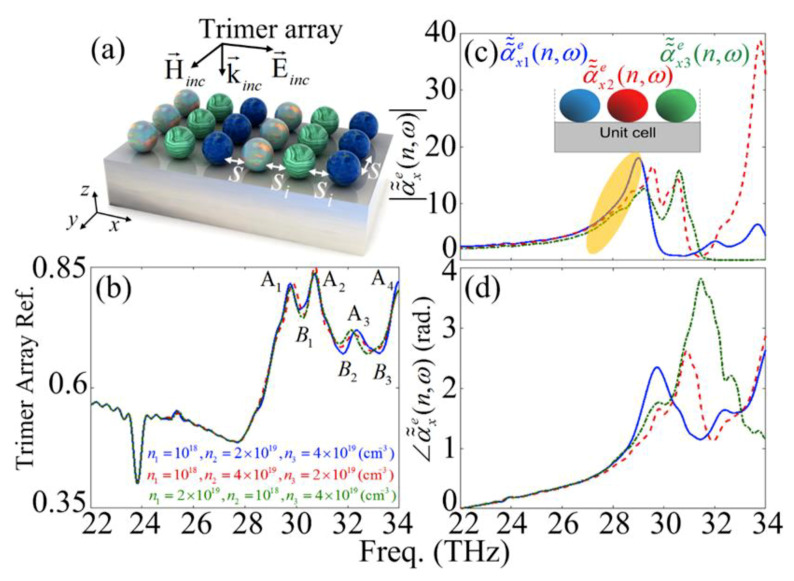
Schematic representation of the square patterned array composed of the SiC dimers (**a**) and trimers (**c**) with different doping values, respectively. (**b**); (**d**) $n_{1}=10^{18}$, $n_{2}=2\times 10^{19}$, and $n_{3}=4\times 10^{19}$ $\left(cm^{-3}\right)$ (solid curve), $n_{1}=10^{18}$, $n_{2}=4\times 10^{19}$, and $n_{3}=2\times 10^{19}$ $\left(cm^{-3}\right)$ (dashed curve) and $n_{1}=2\times 10^{19}$, $n_{2}=10^{18}$, and $n_{3}=4\times 10^{19}$ $\left(cm^{-3}\right)$ (dashed-dotted curve), respectively.

The reflection spectrum bandwidth can be enhanced by increasing the number of interacting dipoles with a different carrier concentration. To further improve and broaden the reflection spectrum of the considered structure, we use trimer cells in the square periodic configuration. Figure 6a demonstrates the schematic representation of the SiC nanoparticle periodic array configuration composed of the linear trimers. The internal separation distance between the nanoparticles and the cell-separation distance between an individual trimer cell with its adjacent cells in the *x*- and *y*-directions are defined as $s_{i}$ and of s, respectively. It should be noted that, we have assumed equal separation distances of $s_{i}=s=$200 nm for the trimer structure. Moreover, Figure 6b depicts the reflection spectrum of the trimer arrays for $n_{1}=10^{18}$, $n_{2}=2\times 10^{19}$ and $n_{3}=4\times 10^{19}$ $\left(cm^{-3}\right)$ (solid curve), $n_{1}=10^{18}$, $n_{2}=4\times 10^{19}$, and $n_{3}=2\times 10^{19}$ $\left(cm^{-3}\right)$ (dashed curve) and $n_{1}=2\times 10^{19}$, $n_{2}=10^{18}$, and $n_{3}=4\times 10^{19}$ $\left(cm^{-3}\right)$ (dashed-dotted curve), respectively.

Accordingly, four separate reflection peaks and three dips known as $A_{1}$–$A_{4}$ modes; and $B_{1}$–$B_{3}$ modes, can be distinguished, respectively. It can be seen that the maximum reflection occurs at $A_{1}$, $A_{2}$ and $A_{4}$ modes. To investigate the origin of the peaks and dips we studied the near-field interaction between the nanoparticles of each trimer unit-cell. Figure 6c,d presents the amplitude and phase of the effective electric susceptibilities of the doped SiC trimer square array for $n_{1}=$ $10^{18}$, $n_{2}=$ $2\times 10^{19}$, and $n_{3}=$ $4\times 10^{19}$ $\left(cm^{-3}\right)$ (solid curve), $n_{1}=$ $10^{18}$, $n_{2}=$ $4\times 10^{19}$, and $n_{3}=$ $2\times 10^{19}$ $\left(cm^{-3}\right)$ (dashed curve) and $n_{1}=$ $2\times 10^{19}$, $n_{2}=$ $10^{18}$, and $n_{3}=4\times 10^{19}$ $\left(cm^{-3}\right)$ (dashed-dotted curve), respectively. It can be seen that at each mode, a pair of in-phase interacting dipoles are interacting with another out-of-phase dipole. Throughout this manuscript the dipolar interactions with very small phase differences [around zero radian] are assumed as in-phase interactions and the interactions with phase difference of around or more than 0.5 (rad.) are assumed as out-phase interactions. For instance, at the $A_{1}$ and $A_{2}$ modes, ${\tilde{\tilde{\alpha }}}_{x2}^{e}$ and ${\tilde{\tilde{\alpha }}}_{x3}^{e}$ pair interact in-phase together and out-of-phase with ${\tilde{\tilde{\alpha }}}_{x1}^{e}$ [with phase differences of 0.4 and 0.7 (rad), respectively]. In contrast, at the $A_{3}$ and $A_{4}$ modes the pair of ${\tilde{\tilde{\alpha }}}_{x1}^{e}$ and ${\tilde{\tilde{\alpha }}}_{x2}^{e}$ are in-phase together and out-of-phase with ${\tilde{\tilde{\alpha }}}_{x3}^{e}$. The same interacting rule governs the $B_{1}$–$B_{3}$ modes. The interaction mechanism of the effective polarizabilities at each mode is summarized in Table 1. Furthermore, according to Figure 6c, it can be seen that in addition to the resonant peaks due to the near-field interactions, the individual resonant peak of the each single nanoparticle is similar to the periodic dimer structure (see the yellow-colored region).

**Figure 7:**
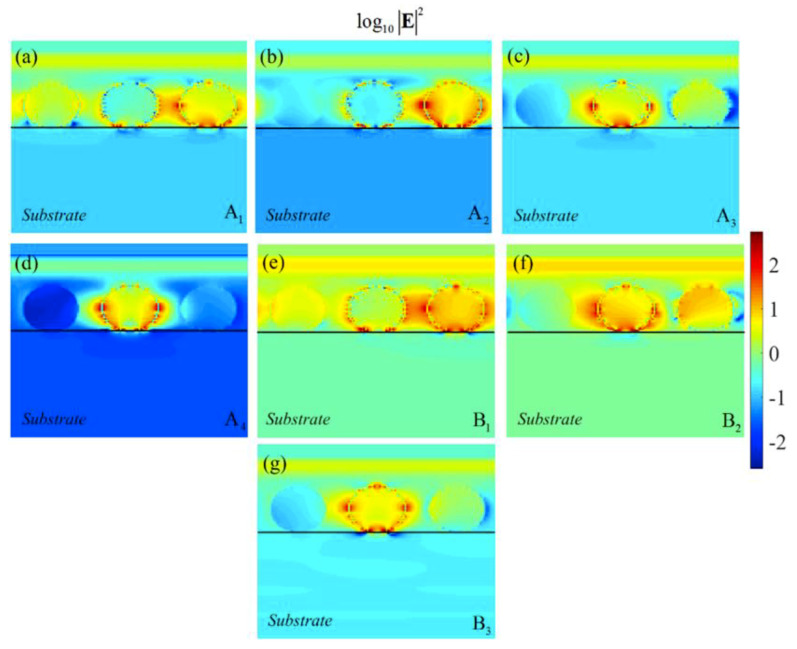
Electric field intensity in logarithmic scale for the periodic trimer array metasurface composed of doped SiC nano-spheres with the doping concentration of $n_{1}=1\times 10^{18}$, $n_{2}=2\times 10^{19}$, and $n_{3}=4\times 10^{19}$ $\left(cm^{-3}\right)$ at (**a**–**d**) the higher reflective modes of $A_{1}$, $A_{2}$, $A_{3}$, $A_{4}$; and (**e**–**g**) the lower reflective modes of $B_{1}$, $B_{2}$, and $B_{3}$, respectively.

Particularly for ${\tilde{\tilde{\alpha }}}_{x2}^{e}$ (dashed curve) and ${\tilde{\tilde{\alpha }}}_{x3}^{e}$ (dashed-dotted curve), these resonant peaks appear in the form of semi-shoulder resonances. Figure 7a–g elucidate the electric field distribution in the *xz*-plane and in a logarithmic scale of the unit-cell of the trimer array metasurface composed of the SiC nanoparticles at $A_{1}$–$A_{4}$ (Figure 7a–d); and $B_{1}$–$B_{3}$ (Figure 7e–g), respectively.

In Figure 7a–g, the carrier concentration for the individual nanoparticles are considered as an $n_{1}=1\times 10^{18}$, $n_{2}=2\times 10^{19}$, and $n_{3}=4\times 10^{19}$ $\left(cm^{-3}\right)$ arrangement. It can be seen that at the $A_{1}$–$A_{4}$ modes the reflection of the incident field is maximized while at the $B_{1}$–$B_{3}$ modes the reflection is smaller, respectively. If the spheres are replaced by other geometrical shapes, such as rods, it enables the excitation of the higher orders of polar resonances using rod size and aspect ratio in a more controlled manner. As the higher order of polar near-field interaction between the neighboring rods may not be as strong as the dipolar interaction, the reflection spectrum from a metasurface composed of rods may be more limited as it is mostly dominated by dipolar interactions.

## 4. Conclusions

In this study, spectral characteristics of SiC metasurfaces are demonstrated using Mie theory. Our results in the first part show how the extra carriers affect the LSPhR and the dipolar behavior of an individual SiC nanoparticle. In the second part, using the FDTD method we numerically demonstrate the spectral broadening of the reflectivity using patterned SiC-doped structures. We have shown that each SiC nanoparticle can be modeled by a resonant electric dipole in the Reststrahlen band (i.e., the negative permittivity region) and a magnetic dipole at the TO phonon frequency. Furthermore, we have demonstrated that enhancing the carrier concentration amounts in a subwavelength nanoparticle leads to a red-shift of the resonant peak in the electric dipole, while the negligible magnetic dipole may not be influenced by the doping. Based on these results we have presented an APEC array configuration of the *n*-doped SiC nano-spheres with a constant radius which can compensate for the reflection amplitude for the frequencies greater than *ω_LO_*_._

## Figures and Tables

**Table:** 

Δφ(rad)	$A_{1}$	$A_{2}$	$A_{3}$	$A_{4}$
${\tilde{\tilde{\alpha }}}_{x1}^{e}$	0.4	1	In-phase	In-phase
${\tilde{\tilde{\alpha }}}_{x2}^{e}$	In-phase	In-phase		
${\tilde{\tilde{\alpha }}}_{x3}^{e}$			1	1.7

**Table:** 

Δφ (rad)	$B_{1}$	$B_{2}$	$B_{3}$
${\tilde{\tilde{\alpha }}}_{x1}^{e}$	0.7	In-phase	In-phase
${\tilde{\tilde{\alpha }}}_{x2}^{e}$	In-phase		
${\tilde{\tilde{\alpha }}}_{x3}^{e}$		2.4	0.6
